# Novel perspectives on early diagnosis of acute compartment syndrome: the role of admission blood tests

**DOI:** 10.1186/s10195-024-00800-3

**Published:** 2024-11-13

**Authors:** Tao Wang, Yubin Long, Qi Zhang

**Affiliations:** 1 Department of Lower Limb Trauma, Beijing Jishuitan Hospital, Guizhou Hospital, Baiyun District, Guiyang, Guizhou China; 2 Department of Orthopaedic Surgery, Third Hospital of Hebei Medical University, Shijiazhuang, Hebei People’s Republic of China; 3 Department of Anesthesiology, Hebei Children’s Hospital, No133 Jianhua South Street, Shijiazhuang, Hebei China

**Keywords:** Acute compartment syndrome, Complete blood count, Derived inflammatory indicators, Tibial fractures, Propensity score matching, Nomogram prediction model

## Abstract

**Purpose:**

The role of admission blood indicators in patients with acute compartment syndrome (ACS) remains debated. Our primary purpose was to observe variations of admission blood indicators in patients with ACS, while our secondary goal was to explore potential biomarkers related to ACS.

**Methods:**

We collected information on patients with tibial fracture between January 2013 and July 2023, and divided them into ACS and non-ACS groups. Propensity score matching (PSM) analysis was performed to lower the impact of potential confounding variables such as demographics and comorbidities. Admission blood indicators were analyzed using univariate, logistic regression, and receiver operating characteristic (ROC) curve analyses. Then, we established a nomogram prediction model by using R language software.

**Results:**

After propensity PSM analysis, 127 patients were included in each group. Although numerous blood indicators were found to be relevant to ACS on univariate analysis, logistic regression analysis showed that monocytes (MON, *p* = 0.015), systemic immune-inflammation index (SII, *p* = 0.011), and creatine kinase myocardial band (CKMB, *p* < 0.0001) were risk factors for ACS. Furthermore, ROC curve analysis identified 0.79 × 10^9^/L, 1082.55, and 20.99 U/L as the cut-off values to differentiate ACS patients from patients with tibial fracture. We also found that this combination had the highest diagnostic accuracy. Then, we constructed a nomogram prediction model with AUC of 0.869 for the prediction model, with good consistency in the correction curve and good clinical practicality by decision curve analysis.

**Conclusions:**

We found that the levels of MON, SII, and CKMB were related to ACS and may be potential biomarkers. We also identified their cut-off values to separate patients with ACS from those with tibial fracture, helping orthopedists promptly evaluate and take early measures. We established a nomogram prediction model that can efficiently predict ACS in patients with tibial fracture.

## Introduction

Acute compartment syndrome (ACS), a common and intractable complication,occurs in 2–9% of patients with tibial fracture [1]. Rapidly increasing intracompartmental pressure and soft tissue edema caused by high-energy injuries can reduce capillary blood flow and tissue *p*O_2_ [2–4]. Protracted diagnosis and treatment may lead to catastrophic outcomes such as muscle necrosis, neurological deficits, contracture, or amputation [5, 6]. Currently, it is a challenge for orthopedists to diagnose ACS promptly, because its diagnosis is commonly based on the experience of orthopedists rather than gold-standard tests.

Ongoing evidence has reported investigations of the predictors of ACS and found that young or male patients and high-energy injuries are relevant to ACS [7–10]. However, to the best of our knowledge, existing literature has predominantly focused on the characteristics of ACS patients, ignoring the important role of admission blood indicators in its diagnosis because tissue ischemia caused by ACS can result in some changes in patients, such as immune indicators [11, 12]. In contrast, our study mainly observed changes in admission blood indicators and then assessed possible biomarkers of ACS.

## Patients and methods

### Ethics statement

Before data collection, this study was approved by the institutional review boards of our hospital (S2020-024-1) in compliance with the Declaration of Helsinki, and an exemption from informed consent was obtained. All data were anonymized before analysis to safeguard patient privacy.

### Patients

We collected data from patients with ACS after tibial fracture at our hospital between January 2013 and July 2023. Patients with tibial fracture were separated into ACS and non-ACS groups on the basis of the occurrence of ACS, which was defined as ∆*P* < 30 mmHg (∆*P* is the diastolic arterial pressure − intracompartmental pressure). The inclusion criteria were as follows: (1) closed tibial fracture, (2) age ≥ 18 years, and (3) no comorbidities. The following individuals were excluded from consideration: (1) those who died while receiving hospital care, (2) those who suffered numerous vascular injuries, (3) those who had pathological fracture, and (4) those with insufficient data.

In the present study, the patient characteristics, comorbidities, and admission laboratory results were obtained. Demographic characteristics included age, sex, body mass index, injury mechanism, length of time from injury to admission, alcohol and tobacco use, and smoking. The comorbidities included heart disease, hypertension, diabetes, and cerebral infarction. We collected the laboratory indicators of patients when they arrived in the emergency department, including hemoglobin (HGB), lymphocyte (LYM), monocyte (MON), neutrophil (NEU), neutrophil-to-lymphocyte ratio (NLR), monocyte-to-lymphocyte ratio (MLR), platelet-to-lymphocyte ratio (PLR), systemic immune-inflammation index (SII), systemic inflammation response index (SIRI), platelet (PLT), white blood cell (WBC), red blood cell (RBC), total protein (TP), albumin (ALB), globulin (GLOB), ALB/GLOB, creatine kinase (CK), creatine kinase myocardial band (CKMB), CKMB%, Ca^2+^, K^+^, Na^+^, Mg^2+^, P^3+^, lactic dehydrogenase (LDH), osmotic pressure (OSM), and glucose (GLU). The derived inflammatory indicators were calculated as follows:

- NLR = neutrophil count/lymphocyte count;
- MLR = monocyte count/lymphocyte count;
- PLR = platelet count/lymphocyte count;
- SII = (platelet count × neutrophil count)/lymphocyte count;
- SIRI = (monocyte count × neutrophil count)/lymphocyte count.

### Statistics

We used SPSS (version 27.0; SPSS Inc., Chicago, IL) and regarded *p* < 0.05 as statistical significance. Because the continuous variables did not meet normality criteria in our study, we used the Mann–Whitney *U* test. For count data, we used the chi-squared test. We performed propensity score matching (PSM) analysis with a 1:1 ratio to adjust for discrepancies in baseline characteristics between the two groups to reduce selection bias and other confounding factors. PSM was determined by logistic regression analysis utilizing covariates such as patient characteristics and comorbidities. After PSM, we investigated the association between blood test results at admission and ACS using univariate and logistic regression analyses. Finally, R language software was used to establish the nomogram prediction model for ACS, then we assessed the discriminative ability of the prediction model on the basis of the AUC of the ROC, and we evaluated the predicted and actual probabilities of this prediction model on the basis of the calibration curve. Decision curve analysis was used to evaluate the clinical application value of the prediction model.

## Results

**Fig. 1:**
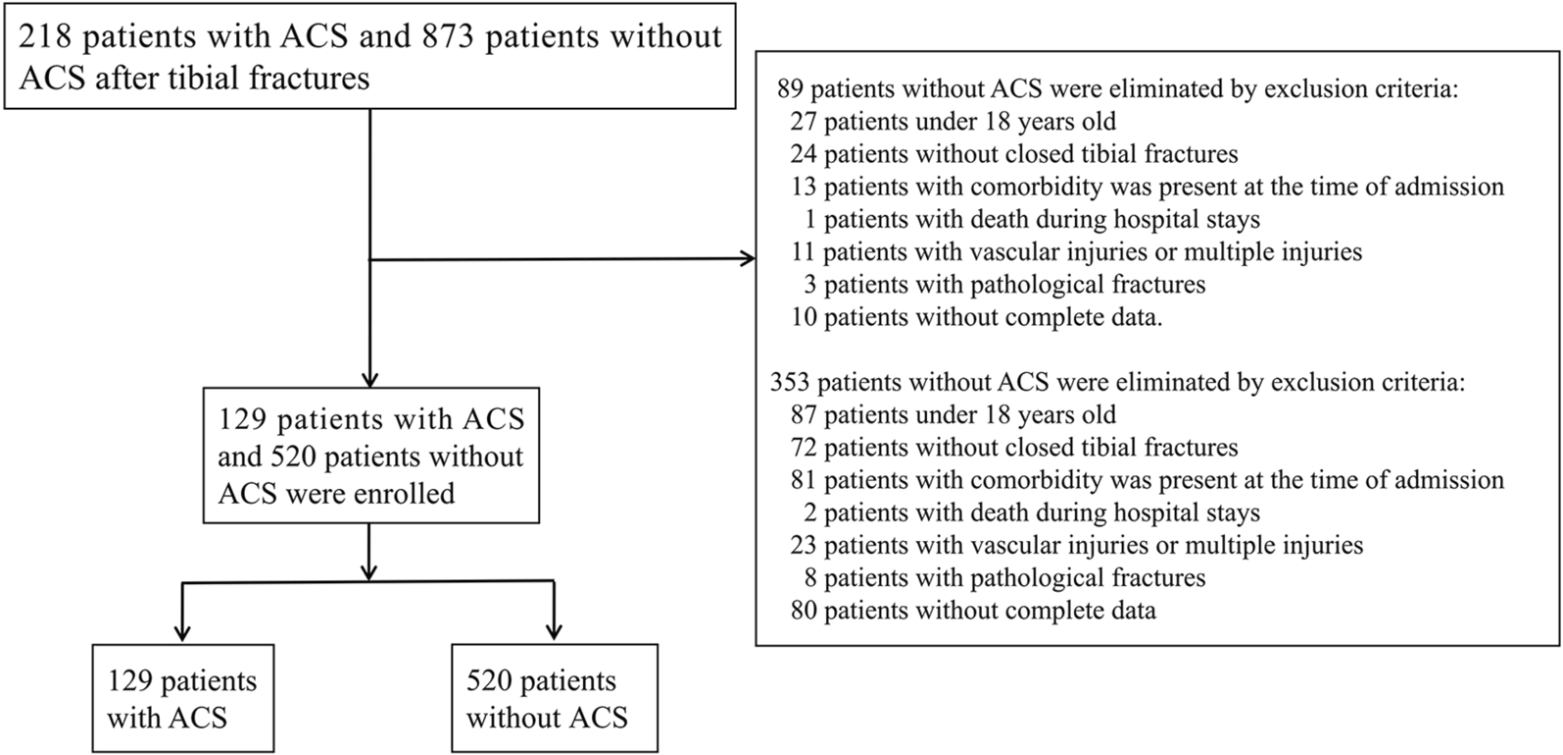
Flow diagram of included patients

A total of 1091 patients with tibial fracture, including 218 with ACS and 873 without ACS, from January 2013 to July 2023, were screened in this study. After selection based on the exclusion criteria, 89 patients with ACS and 353 patients without ACS were excluded (Fig. 1). Finally, 129 patients with ACS and 520 non-ACS patients were included in the study. Table 1 provides an overview of the patients’ baseline characteristics in each group. Before PSM, significant differences were observed in terms of age (*p* = 0.005), sex (*p* < 0.001), crush injury (*p* = 0.001), and history of diabetes (*p* = 0.036). The baseline characteristics of the patients did not change significantly after PSM (all *p* > 0.05).

**Table 1:** Patient characteristics at baseline

Variable	Pre matching			Post matching		
	ACS (*n* = 129)	Non-ACS (*n* = 520)	*p*	ACS (*n* = 127)	Non-ACS (*n* = 127)	*p*
Age, years	35.0 (28.0–48.0)	42.0 (32.0–52.0)	0.005	35.0 (28.0–48.0)	40.0 (33.0–47.0)	0.215
Gender						
Male	120	377	** < 0.001**	118	117	0.811
Female	9	143		9	10	
Body mass index (kg/m^2^)	24.3 (22.9–26.8)	25.0 (22.9–26.3)	0.712	24.3 (22.9–26.8)	25.0 (23.7–26.9)	0.183
Mechanism of injury						
Car crash injury			0.099			0.472
Yes	32	168		30	35	
No	97	352		97	92	
Fall injury			0.400			0.336
Yes	36	165		34	41	
No	93	355		93	86	
Crush injury			**0.001**			0.391
Yes	38	88		36	30	
No	91	432		91	97	
Hurt by a crashing object			0.431			0.427
Yes	29	99		27	22	
No	100	412		100	105	
Time from injury to admission (h)	6.0 (4.0–10.0)	5.0 (3.0–11.8)	0.087	6.0 (4.0–10.0)	5.0 (3.0–10.0)	0.327
Smoking			0.982			1.000
Yes	19	77		17	17	
No	110	443		110	110	
Alcohol			0.540			0.255
Yes	15	51		13	8	
No	114	469		114	119	
Comorbidities						
Heart disease			0.586			1.000
Yes	5	16		3	3	
No	124	504		124	124	
Hypertension			0.055			1.000
Yes	9	68		9	9	
No	120	452		118	118	
Diabetes			**0.036**			0.498
Yes	2	32		2	0	
No	127	488		125	127	
Cerebral infarction			0.392			1.000
Yes	3	6		1	1	
No	126	514		126	126	

The bold value just means significant value

**Fig. 2:**
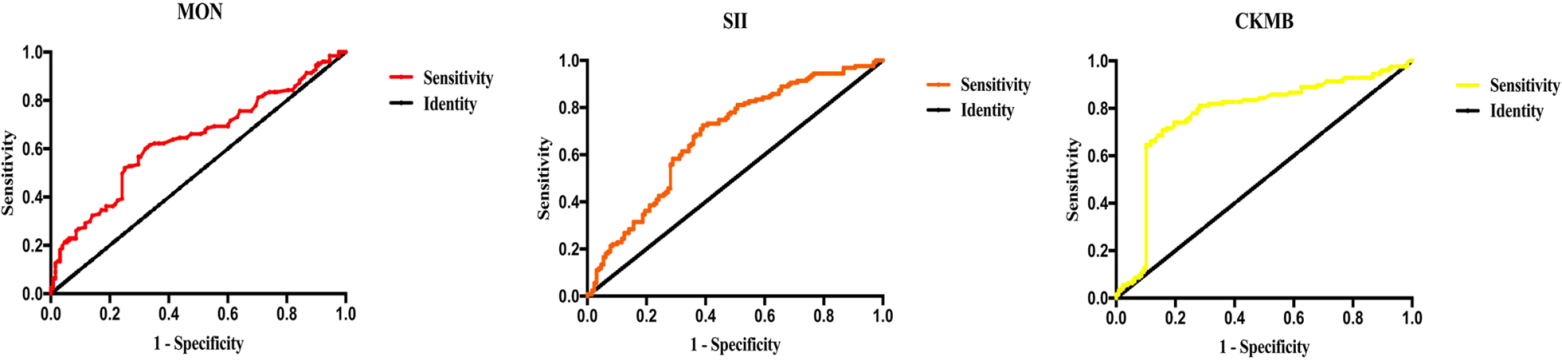
Risk factors of acute compartment syndrome in receiver operating characteristic curve analysis

Table 2 shows that the levels of MON, NEU, NLR, MLR, SII, SIRI, WBC, TP, ALB, A/G, LDH, CK, CKMB, CKMB%, Na^+^, Ca^2+^, Mg^2+^, and GLU showed a significant difference between the two groups (all *p* < 0.05). Furthermore, we performed logistic regression analysis and found that the levels of MON [*p* = 0.015, OR = 3.352, 95% CI (1.266, 8.873)], SII [*p* = 0.011, OR = 1.001, 95% CI (1.000, 1.002)] and CKMB [*p* < 0.001, OR = 1.097, 95% CI (1.071, 1.124)] were independent risk factors for ACS (Table 3). ROC curve analysis showed that the levels of MON [*p* < 0.0001, AUC = 0.632, 95% CI (0.566, 0.700)], SII [*p* < 0.0001, AUC = 0.678, 95% CI (0.612, 0.744)], CKMB [*p* < 0.0001, AUC = 0.865, 95% CI (0.817, 0.913)] were independent risk factors of ACS. We also identified the cutoff values of MON, SII, and CKMB to separate ACS from tibial fracture as 0.79 × 10^9^/L, 1082.55, and 20.99 U/L, respectively (Table 4; Fig. 2).

**Table 2:** Laboratory variables associated with acute compartment syndrome on univariate analysis

Laboratory variable	ACS (*n* = 127)	Non-ACS (*n* = 127)	*p*
Hemoglobin (HGB, g/L)	133.3 (119.7–147.8)	132.1 (123.4–142.2)	0.592
Lymphocyte (LYM, 10^9^/L)	1.53 (1.10–1.92)	1.42 (1.07–1.81)	0.371
Monocyte (MON, 10^9^/L)	0.89 (0.61–1.07)	0.70 (0.54–0.89)	< 0.0001
Neutrophil (NEU, 10^9^/L)	11.57 (8.15–13.82)	6.84 (5.54–8.87)	< 0.0001
NLR	7.05 (5.45–10.96)	4.92 (3.31–7.60)	< 0.0001
PLR	131.28 (111.06–198.95)	140.06 (111.06–196.88)	0.949
MLR	0.55 (0.42–0.88)	0.49 (0.36–0.66)	0.006
SII	1518.81 (1021.6–2306.18)	980.8 (611.53–1672.89)	< 0.0001
SIRI	6.35 (3.56–11.11)	3.63 (2.04–5.37)	< 0.0001
Platelet (PLT, 10^9^/L)	215.53 (183.00–248.00)	213.33 (178.00–269.06)	0.237
Red blood cell (RBC, 10^12^/L)	4.26 (3.81–4.71)	4.18 (4.02–4.61)	0.324
White blood cell (WBC, 10^9^/L)	14.32 (10.87–16.9)	9.37 (8.05–10.30)	< 0.0001
Total protein (TP, g/L)	59.92 (59.92–61.5)	65.18 (59.45–68.54)	< 0.0001
Albumin (ALB, g/L)	37.08 (37.08–40.0)	41.90 (39.2–45.00)	< 0.0001
Globulin (GLOB, g/L)	22.33 (22.20–22.33)	22.30 (18.72–25.01)	0.927
A/G	1.73 (1.60–1.73)	1.90 (1.60–2.20)	0.003
Lactic dehydrogenase (LDH, U/L)	474.78 (474.78–500.00)	200.00 (169.00–252.27)	< 0.0001
Creatine kinase (CK, U/L)	3291.99 (521.00–3291.99)	351.5 (184.6–711.00)	< 0.0001
Creatine kinase myocardial band (CKMB, U/L)	43.88 (30.00–43.88)	16.0 (12.40–22.44)	< 0.0001
CKMB%	7.15 (3.60–7.15)	4.34 (2.95–7.40)	0.017
K^+^ (mol/L)	3.87 (3.81–3.87)	3.91 (3.69–4.15)	0.092
Na^+^ (mol/L)	137.38 (137.38–137.93)	139.3 (137.9–141.16)	< 0.0001
Ca^2+^ (mol/L)	2.14 (2.14–2.20)	2.21 (2.12–2.31)	< 0.0001
Mg^2+^ (mol/L)	0.79 (0.77–0.79)	0.86 (0.79–0.90)	< 0.0001
P^3+^(mol/L)	1.09 (1.08–1.10)	1.09 (0.93–1.28)	0.773
Glucose (GLU, mmol/L)	7.86 (7.15–7.86)	5.68 (5.17–6.43)	< 0.0001
Osmotic pressure (OSM, umol/L)	270.4 (270.4–270.7)	270.6 (267.6–274.5)	0.390

*NLR* neutrophil-to-lymphocyte ratio, *MLR* monocyte-to-lymphocyte ratio, *PLR* platelet-to-lymphocyte ratio, *SII* systemic immune-inflammation index, *SIRI* system inflammation response index

**Table 3:** Logistic regression analysis of laboratory variables associated with acute compartment syndrome

Variable	OR	95% CI		*p* value
		Lower limit	Upper limit	
MON	3.352	1.266	8.873	0.015
SII	1.001	1.000	1.002	0.011
CKMB	1.097	1.071	1.124	< 0.001

*MON* monocytes, *SII* systemic immune-inflammation index, *CKMB* creatine kinase myocardial band, *FIB* fibrinogen

**Table 4:** ROC curve analysis and cutoff values associated with acute compartment syndrome

Variable	Area	*p*-value	95% CI		Cutoff value
			Lower limit	Upper limit	
MON	0.632	< 0.0001	0.566	0.700	0.79 10^9^/L
SII	0.678	< 0.0001	0.612	0.744	1082.55
CKMB	0.865	< 0.0001	0.817	0.913	20.99U/L

*MON*  monocytes, *SII* systemic immune-inflammation index, *CKMB* creatine kinase myocardial band

**Fig. 3:**
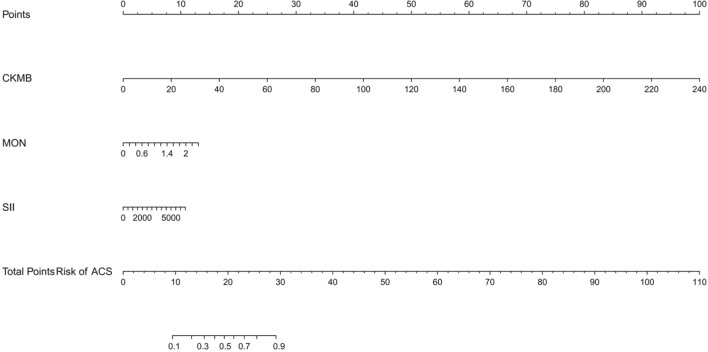
The nomogram prediction model for acute compartment syndrome

**Fig. 4:**
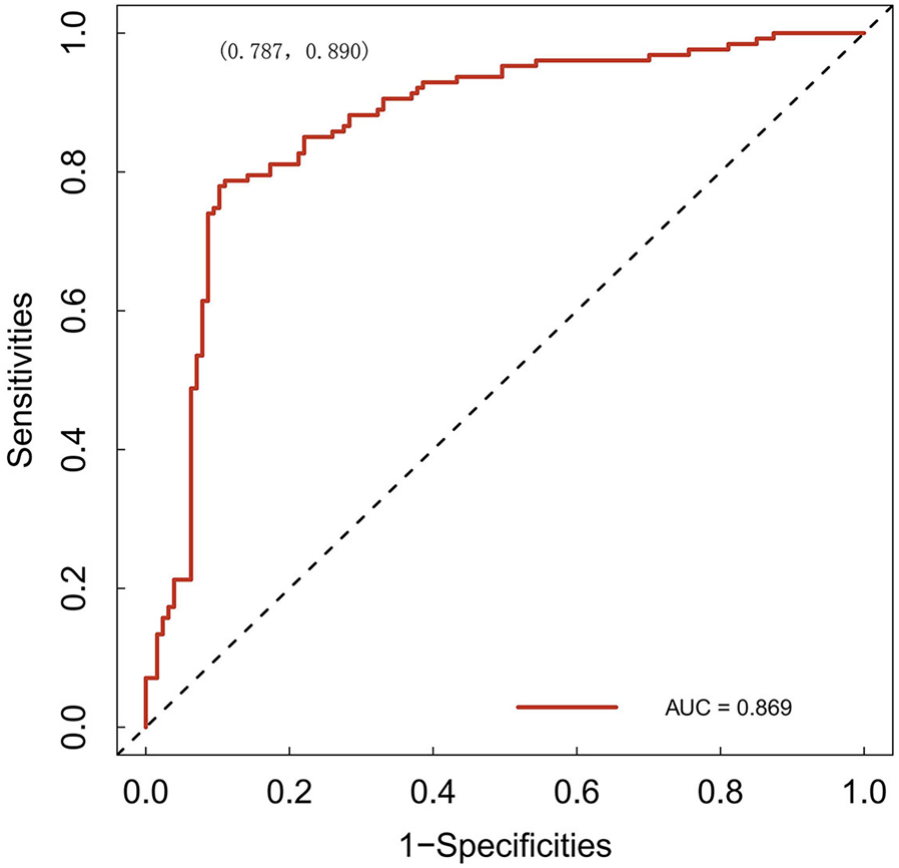
The receiver operating characteristic curves of the nomogram

**Fig. 5:**
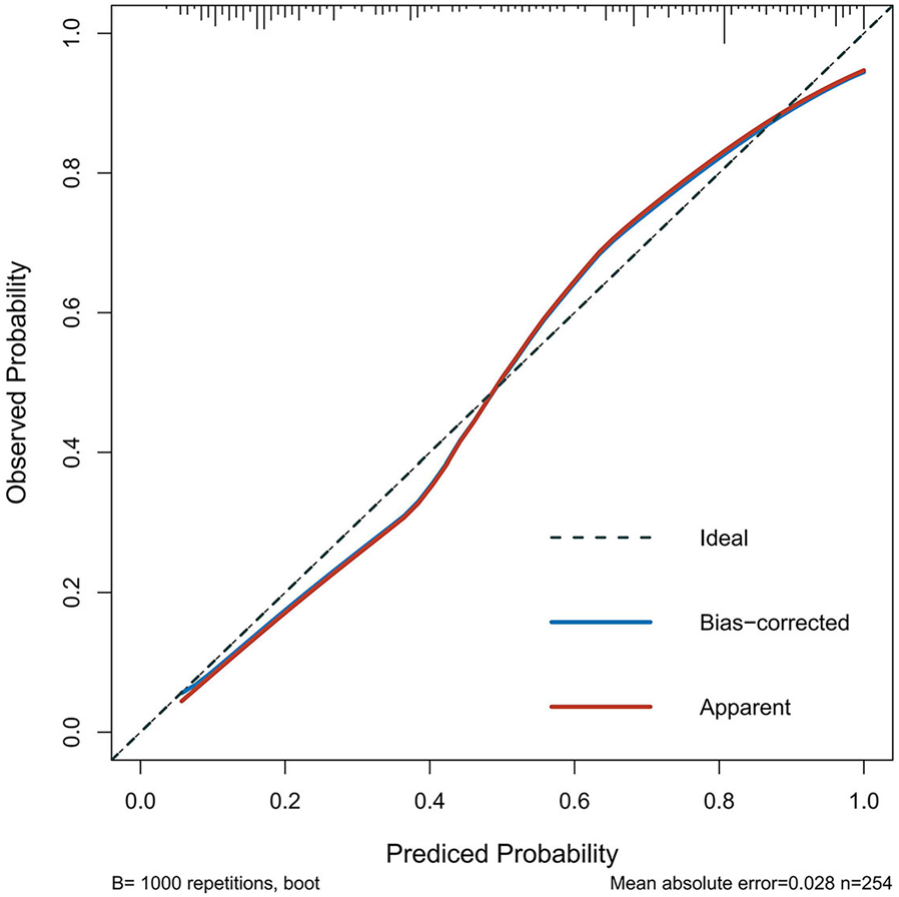
The calibration curve of the nomogram

**Fig. 6:**
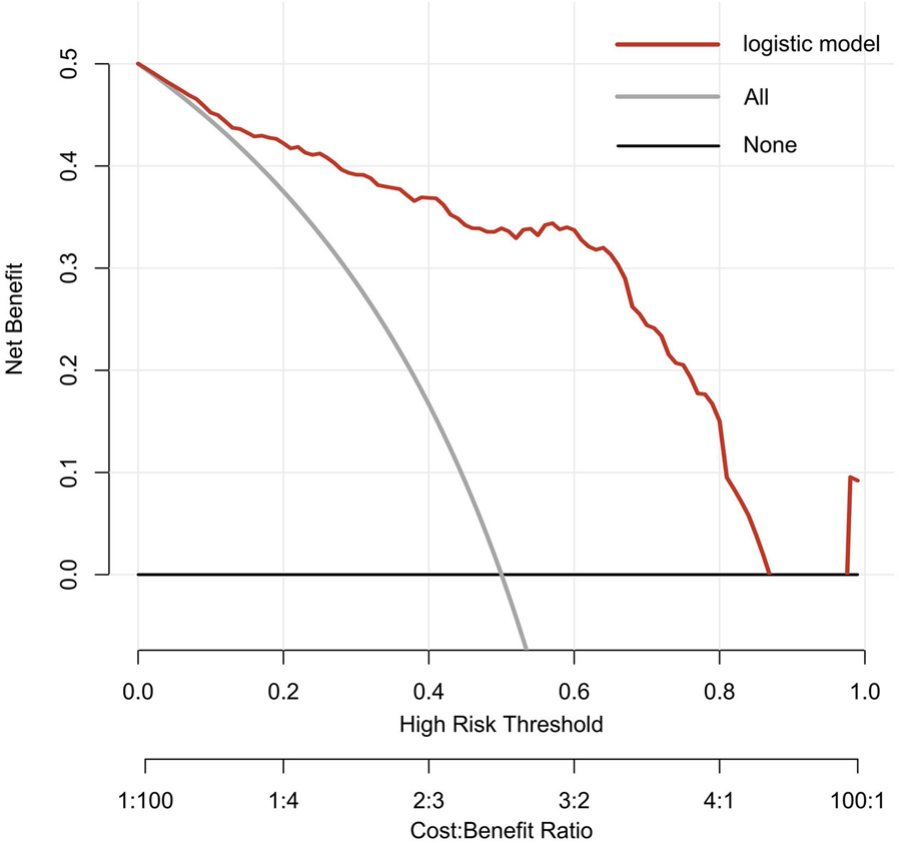
The decision curve analysis of the nomogram

As shown in Fig. 3, we conducted a nomogram prediction model based on logistic regression analysis. As present in Figs. 4 and 5, the ROC curve of the nomogram suggested good discrimination ability [AUC = 0.869, 95% CI (0.787, 0.890)], and the calibration curve of the nomogram with the Hosmer–Lemeshow goodness-of-fit test showed good calibration (*p* > 0.05). Decision curve analysis (Fig. 6) showed that our nomogram prediction model exhibited good clinical benefits.

## Discussion

ACS, which commonly occurs after fracture, can cause tissue ischemia owing to increased intracompartment pressure [13–15]. It often results in catastrophic complications such as infection, amputation, or muscle necrosis. In recent decades, increasing evidence has been reported regarding the incidence and predictors of ACS. Our meta-analysis found that being younger or male and injury mechanisms were related to ACS [1]. After reviewing the relevant articles, prior research focused on the characteristics of patients with ACS, but neglected the role of admission blood indicators. Therefore, we investigated their role in the development of ACS by using PSM to minimize selection bias and other confounding effects.

In our study, numerous laboratory indicators were found to be relevant to ACS on univariate analysis; however, MON, SII, and CKMB levels were risk factors for ACS on logistic regression analysis. ROC curve analysis indicated that 0.79 × 10^9^/L, 1082.55, and 20.99 U/L were the cut-off values for predicting ACS. Then, we constructed a nomogram prediction model with an AUC of 0.869, with good consistency in correction curve and good clinical practicality by decision curve analysis.

It is well known that the hypoxic and ischemic microenvironment caused by continuously increasing intracompartmental pressure can lead to aseptic inflammation [11, 16–18]. Therefore, the presupposition of an inflammatory mechanisms in the pathogenesis of ACS is highly reliable. Blood biomarker research is the most commonly used method owing to its routine collection and low cost, which can enable large-scale practical and clinical applications. A complete blood count test, including NEU, MON, LYM, and platelets, which are inexpensive, can be easily obtained and are sensitive to inflammation in the organism [19].

NEU is the most abundant type of WBC in the body and contributes greatly to the innate immune system [20]. LYM is an adaptive immune system that contributes to immunological responses, including antibody generation and cell-mediated immunity [21]. MON is the most critical cell type for releasing proinflammatory and prooxidant cytokines during the innate immune response against pathogens [22]. PLT have the potential to modulate endothelial cell permeability, as well as the recruitment of NEU and macrophages, and has inflammatory roles in various pathophysiological conditions [23]. There has been increasing interest in inflammatory ratios, including NLR, MLR, and PLR, which are derived from a complete blood count, being a low-cost and routine clinical examination [24]. Inflammatory indicators derived from NEU, MON, LYM, and PLT, such as NLR, MLR, PLR, SII, and SIRI, are new markers that reflect immune response and inflammation [25, 26].

The SII, which combines NEU, LYM, and PLT, is becoming a popular biomarker for systemic inflammation [27] because it can more precisely represent the immunological and inflammatory conditions of the body than any of the aforementioned markers alone. Previous research has shown that SII plays a key role in determining the prognosis of many physical disorders, including malignancies, cerebral infarction, cardiovascular disease, and acute pancreatitis. SIRI is a novel inflammation-based biomarker that combines NUE, LYM, and MON peripheral counts [26]. Previous research has shown that SIRI can represent inflammation and has prognostic value in various malignancies, including cholangiocarcinoma and esophageal and gastric cancers [28]. To the best of our knowledge, the relationship between these indices and ACS has not been studied.

Univariate analysis showed that MON, NEU, NLR, MLR, SII, SIRI, and WBC count were associated with ACS. Furthermore, logistic regression analysis indicated that MON and SII levels were independent risk factors for ACS, and ROC analysis showed that the area under the curve of MON and SII was 0.632 and 0.678, respectively, indicating that SII more accurately reflected the immunological and inflammatory conditions of the body than MON. The above-mentioned findings imply that patients with ACS are in an inflammatory state compared with patients without ACS following tibial fracture. This further verifies our previous conclusions on the changes in the proportions of MON and its derived cell type, macrophages, in ACS patients from a study focusing on the deep fascia with single-cell RNA-seq analysis [11]. We will pay great attention to the study of inflammatory biomarkers of ACS using multiomics. Additionally, we identified the cutoff values of MON and SII to predict ACS, which assists orthopedists in auxiliary diagnosis of ACS in clinical practice.

Owing to their excellent sensitivity and specificity, CK and CKMB have frequently been used in clinical practice to diagnose acute myocardial damage [29]. They have also been used to monitor other injuries such as skeletal muscle injury [30], pulmonary embolism [31], and brain injury [32]. However, numerous studies have examined the effects of CK and CKMB on the diagnosis of ACS. In this study, we discovered that CK, CKMB, and CKMB% played a critical role in ACS, according to univariate analysis, while CKMB was an important predictor of ACS on the basis of logistic regression analysis. Furthermore, we identified 20.99 U/L as a cutoff value for CKMB to indicate ACS with the highest accuracy as a single indicator.

We established a prediction model to evaluate ACS in patients with tibial fracture. The ROC curve suggested good discrimination ability, and the calibration curve showed good calibration. Decision curve analysis showed that our nomogram prediction model had good clinical benefits.

This study provides some innovative findings; however, a few limitations should be noted. First, the single-center nature of the research and the limited samples make it difficult to perform subgroup analysis, which inevitably affects the reliability of the findings. Therefore, a multicenter, randomized controlled study with a larger sample size is required. Second, the inherent limitation on data collection owing to its retrospective nature results in the omission of some important inflammatory markers, such as C-reactive protein.

In conclusion, we used PSM to reduce the effects of potential confounding variables and investigate the role of admission blood indicators. Our findings showed that MON, SII, and CKMB levels were risk factors for ACS. We also identified their cutoff values. We established a nomogram prediction model that can efficiently predict ACS in patients with tibial fracture.

## Data Availability

Yes.
